# New Injection Method

**Published:** 1881-10

**Authors:** 


### NEW INJECTION METHOD.

Dr. Sesemann made a report for the Deutsche Ærztliche Verein of St. Petersburg, *St. Petersburg Med. Wochenschrift* of various means he has employed for the perservation of the bodies of children and of portions of adult cadavers. His object was to preserve as nearly unchanged as possible the form and color of the parts. The syringes in general use are unsuitable because the fluid is forced in by too great a pressure, bursting some arteries and here and there breaking down the tissues. His plan is to use the irrigating apparatus or douche, the colume of fluid being from one and a half to two metres in height. The natural colors can be retained by the use of only the metallic salts, chloride of zinc and corrosive sublimate in glycerine and alcohol. J. G. H.

